# Mr. Ring, on Inoculation

**Published:** 1806-01

**Authors:** John Ring

**Affiliations:** New Street


### *To the Editors of the Medical and Physical Journal.*

Gentlemen,

In your Journal for November, you observe, that Mr. Birch and Mr. Walker deny they had pronounced the eruption on the child, in Midford Place, to be small-pox, as stated in the preceding number. I could not but be surprised at this assertion, having been assured of the contrary by Mrs. Ball, the mother of the child. I therefore called on her again, and she again assured me that her former declaration was true; adding, that those gentlemen agreed, the eruption could not be any thing but the small-pox, on account of there being the *snuff* in it.

This intrinsic evidence fully convinces me, that they really made such a declaration, whether they recollect it or not. There is no room to doubt, that they pronounced the word *slough* properly; and that Mrs. Ball mistook it for a word with which she was better acquainted. She now says, that when Mr. Walker saw the case the last time, he acknowledged it could not be the small-pox; because because the eruption continued to come out for the space of more than three weeks.

A case was lately published in the Gentleman's Magazine by Dr. Moseley, which appears to me to be unfounded; and which ought not to pass without notice. It is there stated, that a child of Mr. Mazoyer, of Grafton Street, Soho, had the small-pox in a very severe degree, after vaccination. The case proved fatal, as might well be expected by those who saw it; but there is reason to conclude, that the child never had the cow-pock; for Mrs. Mazoyer assures me, that the inoculation *left no mark behind.* This, in the opinion of an antivaccinist, is a circumstance of little importance.

I now beg leave to offer a few additional remarks on the subject of testing those who have undergone vaccination; a subject that has given rise to great misrepresentation; which is not to be wondered at, when we recollect how many persons write on this practice, and how few understand it.

Being the first who inculcated any caution in this respect, it was natural to suppose I should not escape censure. The enemies of vaccination, and those friends of it, who are ignorant what an ordeal it has already undergone, unite in the clamour on this occasion. The truth is, that the test of variolous inoculation is neither so formidable as that it ought never to be made; nor so indifferent, as that it ought to be made upon every occasion.

It is worthy of remark, that among the failures hitherto acknowledged, scarcely any have occurred in consequence of inoculation; so that we have no proof of the superior efficacy of this test. Many false alarms have thus been excited; and some lives have thus been endangered; while the subject has been left still involved in as much obscurity as before. This, it cannot be denied, has, in many cases been owing either to the ignorance or ill design of the parties who made and published the experiments; but at the same time it is an argument of the fallacy and inferiority of the test.

That it was at first necessary to try this test, as a satisfaction to the patient, the practitioner, and the public, no man will hesitate to admit; particularly, as the fallacy of it, and the extent of the danger attending it, were not at that time so well understood. But when so many parents are anxious to try the experiment, it is proper the whole truth should be laid before the public. This I have hitherto embraced every opportunity of doing; and on the present present occasion, wish to diffuse the knowledge of a case extracted from Mr. Dunning's publication, entitled, "Minutes of some experiments to ascertain the permanent security of vaccination."

This case, to which I formerly alluded, has alone been sufficient to deter one mother, a lady of high rank, from the hazardous experiment; and, as a proof that those who make it in the present state of the practice, and those who suffer it to be made, do it from ignorance, not a single parent, or patient, out of many thousands, after a full explanation of the subject, has wished me to employ that test for upwards of four years.

The case which I now bring forward, in addition to several others which I have already communicated, is contained in a letter from Dr. Stewart, to Mr. Dunning, dated August 11, 1804. It occurred in a daughter of Dr. Stewart, who had been inoculated for the small-pox by himself six years before; and had nearly two hundred pustules, which maturated completely. On the 24th of July, 1804, she was again inoculated with variolous matter, which was inserted in a larger quantity than usual; as is commonly done, when any person is put to the test. By the next day, the arm was more inflamed, and the cuticle more elevated, than Dr. Stewart had ever seen in any case, four or five days after inoculation, in ordinary circumstances. On the fifth day, the inflammation was considerably increased. On the sixth, the local affection still appeared to increase, and to be near its height.

The pustule was small, but became prominent rather suddenly, and in some degree resembled a small grain of wheat. Its base was of a light red or pink colour.

Some children who had been vaccinated, were submitted to the same test, at the same time; and the local effect on their arms was the same. On the sixth day, several pimples appeared on each of them; and those on Miss Stewart, who had had the small-pox, were in every respect similar to those on the other children, who had had the cow-pock; but more numerous, amounting to fourteen or sixteen, whereas in the others the number did not exceed six or eight. There was some whiteness on the tongues of all the children, and slight indisposition; rather more apparent in Miss Stewart than in the others.

On the seventh day, the pimples and indisposition were nearly the same; and in most of the arms, the areola extended to nearly the size of a sixpence. On the eighth day it was as large as a shilling, and of a bright vivid rednes ness. A few small acuminated eruptions appeared this day on the wrist of the inoculated arm of one of the children who had undergone vaccination.

On the ninth day, Miss Stewart and one of the other children, were seized with symptoms resembling those of the eruptive fever of the small-pox; and in the former they were much more violent, than when she originally had that disease. The pock, on the arms of all those who had been vaccinated, had much the appearance of the cow-pock; while that of Miss Stewart assumed the appearance of the inoculated small-pox. There were, at this time, one eruption on the left cheek, and three on the upper lip, of one of the other children, approaching more to the nature of the small-pox than any that Dr. Stewart had before seen. Miss Stewart's arm had now a still more vivid inflammation; and more eruptions appeared on her than on the other child. Many of them were acuminated; and one in the face maturated; but the fluid escaped while Dr. Stewart was absent.

On the two following nights these two children were very restless, and had much fever; and during the day-time they continued greatly indisposed. On the tenth day two small eruptions appeared in the face of the child who had been vaccinated, who also had some degree of delirium. The scabbing process in the arm of Dr. Stewart's youngest child was now complete; the effects of the variolous inoculation, in her, having been very inconsiderable. On the eleventh day, matter was taken from the eruptions of the child who had been previously vaccinated, and used in inoculation; but at the time when Dr. Stewart wrote, the result could not be determined.

On the twelfth day, several fresh eruptions appeared on Miss Stewart. On the eighteenth she was much recovered; but, Dr. Stewart says, she certainly suffered more than any child whom he ever inoculated. Her case, he thinks, *clearly points out the danger of introducing morbid matter into the circulation;* and confirms the observation of the immortal Jenner, that the constitution cannot, by previous infection, be rendered totally insusceptible of the variolous poison. "Why, therefore," says Dr. Stewart, "should we expect more from the cow-pox than from the casual or inoculated small-pox?"

He also observes, that from the 29th of July to the date of his letter, a period of thirteen days, there was a succession of eruptions on the two eldest children; which, he observes, is very different from any thing we ever meet with with in the inoculated small-pox. There was likewise, on the finger of Miss Stewart, a pit very much resembling that of the confluent small-pox.

An introductory letter from Drs. Remmett and Woollcombe, to Mr. Dunning, is prefixed to that of Dr. Stewart; in which those gentlemen declare, that nothing in Dr. Stewart's experiments has induced them to alter their opinion respecting vaccination, as expressed in a former letter; and that it is not natural to expect persons who have had the cow-pock to be more insusceptible of variolous infection, than those who have had the small-pox. They observe, that in all these cases, the symptoms were different from those usually attending the inoculation of the small-pox, the inflammation of the arm appearing, and the indisposition and eruption occurring, at an earlier period than usual. This is similar to what I remarked, in the cases published by Mr. Goldson and Dr. Rollo.

Dr. Remmett and Dr. Woollcombe then express their sentiments in the following words. "We cannot conclude without concurring with Dr. Stewart, in wishing *that the test of variolous inoculation may not generally be had recourse to.* For although, in by far the greater number of instances, no inconvenience has resulted from the practice, yet experience in these and other cases sufficiently proves, that *variolous matter cannot always be introduced into the system with impunity. In those cases where it cannot exert its specific power, it is still capable of exerting the pernicious influence of a morbid poison.*"

Mr. Dunning himself also subjoins his own sentiments on this important subject, and confirms the opinion which I had before given in my letter to Mr. Grant, and my answer to Mr. Goldson; an opinion which has provoked the censure of certain persons, who write on a subject they do not understand.—Mr. Dunning says, "If the events which took place in the preceding cases, have not immediately opened to us new sources of information, *they have amply taught us, how unwise it is to introduce morbid poisons into the system.*

In giving these opinions against a continuance of the variolous test, I may again be represented as discouraging experiments. To this I must, in some measure, plead guilty. It is not, however, my wish to discourage experiments, which are either necessary or useful. The test of variolous inoculation was once useful and necessary; but I am not singular in thinking, that it has for a long time past been otherwise; and that it has not only endangered life life in more than one instance, but rather darkened the subject it was intended to elucidate, and spread a general, though false alarm.

At the conclusion of his pamphlet, Mr. Dunning draws a comparison between vaccine and variolous inoculation, as they have appeared in the populous town of Plymouth, highly favourable to the former; which, although it had been practised very extensively above four years, had not occasioned a single death, nor any appearance of danger. On the contrary, in Mr. Dunning's opinion, many obstinate cutaneous diseases had been removed by it; the health of many delicate and apparently diseased children had been improved, and not an individual had been to any degree injured.—On the other hand, for several years before the introduction of this practice, two at least, and some say more, died in every hundred, of those who were inoculated with the small-pox; a great number recovered with difficulty; and many still have to lament lost or impaired sight, scrofulous affections, or shattered constitutions, during life. Among the latter, one of Mr. Dunning's daughters is an example. Mr. Dunning expresses a hope, that if the inoculation of the small-pox should ever be resorted to again, it will not be allowed in large towns; but the subjects of it be obliged to perform a short quarantine.

The following failure in variolous inoculation, among many others which have come to my knowledge, was lately communicated to me by Mr. Tremaine, of Sydenham, who was inoculated by the same practitioner to whom the unfortunate case occurred.—Lady Morris, formerly of Werrington, was inoculated by the late Dr. Colwell, of Bodwin, and declared safe; but she afterwards caught the small-pox at Bath, and died of that disease.

I shall now make a few remarks on the publication which has done so much temporary mischief, but which, I am confident, will ultimately do great service to the cause of vaccination and humanity; it is scarcely necessary to say, that I mean the publication of Dr. Rowley.

The part I have taken in promoting vaccination, left no room to doubt that I must have incurred Dr. Rowley's resentment, and consequently, that in what he was about to write on the subject, I should be honoured with some share of his attention. But I little expected that he would have advanced what is not only untrue, but so improbable, that no person of common sense can believe it.

Of this kind is the story about Daniel Butler, whom I

vaccinated vaccinated at his own request, at a time when he thought he was not infected with the small-pox. Dr. Rowley says, that I vaccinated him, in order to render the small-pox lighter. This, from whatever quarter it originates, is a false assertion; but had I vaccinated him with that view, it is well known to every one who knows any thing of the subject, that I should have been justified by the example of the most eminent members of the medical profession.

In my Treatise on the Cow-pox, I noticed the expression of *insane projects,* and *poisonous experiments,* which occurred in Dr. Rowley's "Cogent Reasons." This expression appeared to be aimed at vaccination as well as caustic bougies; and I preferred the reasoning of Mr. Home and Mr. Blair to that of Dr. Rowley. This was an unpardonable offence, and has given rise to a keen retort in Dr. Rowley's present publication; he there calls the inoculation of this man for the cow-pox, a vaccinating insane project. The anti-vaccinists are like mad Tom, they think all the world mad; and all the world think them so.

But there is a charge of a more serious nature brought against me by Dr. Rowley, namely, that when this man came to me to shew the small-pox, which appeared about the sixth day, "he was violently hastened out of the house; but not before he fainted, and dropped down almost lifeless from fear." This also, from whatever quarter it originates, is a most base, malicious, and impudent falsehood.

Mrs. Butler, the widow of the person above mentioned, positively denies that she gave Dr. Rowley any such information. She states, that when I told him his complaint was the small-pox, he was much affected, and fainted away. In fact, he was much indisposed from the disorder itself; and I had no doubt but he supposed it to be the small-pox, because there was a very copious eruption, till he informed me, that he thought it the effect of vaccination.

In the second edition of his contemptible farrago, Dr. Rowley softens the charge, and says, that "he was violently hastened out of the house. Properly, lest he might have infected patients who came to be vaccinated." This, as far as it is intelligible, for we must not expect any thing very intelligible in the writings of Dr. Rowley, means, that it was proper to hasten a man who had the small-pox violently out of the house, lest he should infect other patients The truth is, that he was not hastened out of the house at all, but every attention was paid to him which his case required. Proper means were used to revive him, and he remained remained in the house as long as he thought proper. No one can suspect that a practitioner could conduct himself with the least inhumanity to an unoffending individual, who had just before placed confidence in him, but a man who is capable of such an outrage. No other patients were present whom he could infect; and we all know, that there is very little infection in the small-pox at its first appearance.

Two circumstances, it is since known, gave offence to Mrs. Butler. The first was, that I declined attending her husband in the small-pox. This I conceived it my indispensible duty to do, on account of the great number of engagements at that time on my hands, particularly as I was attending many children, who were not yet secured from that disorder. The other cause of offence was, the supposed aggravation of the small-pox in this case, by the cow-pock, a venial error in this poor woman, but not so in a medical man.

I had also attempted to vaccinate the child of this patient, but the practice was then in its infancy, and no infection took place. Hearing that the child had escaped the small-pox, though exposed to it during the whole illness of the father, I went to the house as soon as I thought there was no particular danger of bringing away infection and communicating it to others, and recommended that vaccination should be repeated. This Mrs. Butler declined, but she afterwards consented to it, in consequence of the persuasion of the gentleman who had attended Mr. Butler. When I called for this purpose, Mrs. Butler shewed no sort of resentment, which she certainly would have done, had I been guilty of any want of humanity to her husband.

Dr. Rowley says, this man was inoculated by me, when the small-pox was in the house. The truth is, the small-pox was in the house of the landlord; but this patient lived in a back house, where his widow still lives, at No. 42, Marybone-lane, and informed me, that he was in hopes he was not infected. Dr. Rowley tries to persuade his readers, that the experiment probably hastened the death of the patient. This is what he has taken great pains to inculcate, in order to prejudice the public against vaccination, and with some degree of success.

But when he reflects on the nature of this kind of success, he cannot be much elated; and when he reflects on the uncertainty of its continuance, he may think it prudent to act with a little moderation, and not pursue his triumphs too far. He himself tells us, in his present publication, that "the irresolute, superficial, and credulous public, who cannot be judges of medicine, frequently receive ceive the violence of an enthusiast, or the *semblance* of reason, for *reason itself*; and rest perfectly satisfied with the jejune arguments issuing from fanaticism or profound artifice: but penetrating and reasoning minds perceive their fallacy, and resist their seductive power. *The artful erect the structure of their extraordinary success in life on a supposition, that the majority of mankind are absolute fools, credulous ideots, and easily seduced; particularly in every thing, concerning a science they cannot understand.*"

Here it is evident, from Dr. Rowley's own confession, that he is one of those persons, who entertain such a supposition. He is one of those, who think the public ignorant, credulous, and incapable of becoming judges in medicine. He is one of those, who think the majority of mankind "easily seduced." Hence "penetrating and reasoning minds" may, perhaps, be led to suspect, that Dr. Rowley has taken advantage of the weakness and credulity of the public, and "erected the structure of his extraordinary success in life" on that happy basis.

Dr. Rowley speaks with contempt of those branches of the healing art to which he once belonged. He is now, it is true, a Member of the University of Oxford, and a Licentiate of the Royal College of Physicians; he is author of many books, and of many hand-bills, which are stuck up against the walls in every stinking corner of the streets, in order to inform the public, that the Doctor continues to practice his *mild, experienced, safe, successful, certain, and infallible mode of inoculating for the small-pox*, as usual, at his house in Savile Row; and that he is perfectly well acquainted with the treatment of all the new beastly diseases occasioned by the cow-pox. But after this unintelligible jargon, no surgeon, apothecary, or man-midwife can despair of obtaining all the honors, and all the privileges of the medical profession.

In my Treatise on the Cow-pox, I long ago remarked, that the enemies of vaccination are a little unreasonable, in expecting that we should be able to refute false reports, as fast as they can invent them; I shall, however, select a few of those brought forward by Dr. Rowley, which deserve particular notice; and thus enable the public to form a general opinion of the whole.

The first which presents itself to our view, upon opening his book, is the *ox-faced boy*. This is a most *barefaced imposition*. The boy has tumours on his face, which disfigure him, as all tumours ever must do; but he is no more like an ox, than the man who compared him to that animal is like an ass. His

His present complaint did not commence till long after vaccination; it is of a scrofulous nature, and is evidently hereditary. A younger child, who never was vaccinated, had the same affection in the groin a few months ago, after the natural small-pox; of which Dr. Rowley could scarcely be ignorant, since he was attended by the apothecary of the Marybone Infirmary, of which Dr. Rowley is physician.

The cause of this complaint is easily traced to the father of these children, which could not possibly escape the notice of Dr. Rowley, when he frequented the house. The moment I saw this man, and while he was telling me that he had never had any disorder of the kind, I discovered a large and deep scar under the lower jaw. I then inquired what occasioned it. He replied, that when he was about thirteen years old, he kept cows in Hampshire; and one day sat down on the grass when the ground was wet. In consequence of this, he had a number of large swellings all round his neck, one of which suppurated and broke, and did not heal for three years. This is one of the parents whom Dr. Rowley calls *healthful!*

The next object which presents itself in Dr. Rowley's publication, is the girl with cow-pox mange, &c.; but this shall be the subject of a future letter.

Ere I conclude, I beg leave to correct a false report, which has crept into a periodical publication; and if unanswered, may do mischief. It is in the Anti-Jacobin Review, where it is stated, that a case of the small-pox after the cow-pock occurred in the family of the Rev. Mr. Trollop, Master of Christ's Hospital. This is one of the many unfounded reports, which I long ago investigated. Three children of Mr. Trollop were inoculated for the small-pox; and Mr Trollop was informed, that the matter was taken from a child who had previously had the cow-pock. I have inquired into the case, and am assured by Mr. Ridout, the surgeon who attends the family, and by the parents of the child, that the report is totally destitute of foundation.

Mr. Ridout had inoculated three children in Little Britain for the cow-pock; but declared that the operation had proved ineffectual. The parents were of the same opinion; but, anxious to secure their children from the infection of the natural small-pox, they had them inoculated with variolous matter. This was the source from which the virus was procured for the children of Mr. Trollop.

Trollop.—Those who mean well, will, it is hoped, be a little more cautious in future, how they propagate such reports.

I am, &c.

*New Street, Hanover Square.* JOHN RING.

